# Fluorophenol-Containing Hydrogen-Bond Acidic Polysiloxane for Gas Sensing-Synthesis and Characterization

**DOI:** 10.3390/polym14061147

**Published:** 2022-03-13

**Authors:** Michał Grabka, Przemysław Kula, Mateusz Szala, Krzysztof Jasek, Michał Czerwiński

**Affiliations:** Institute of Chemistry, Faculty of Advanced Technologies and Chemistry, Military University of Technology, 00-908 Warsaw, Poland; przemyslaw.kula@wat.edu.pl (P.K.); mateusz.szala@wat.edu.pl (M.S.); krzysztof.jasek@wat.edu.pl (K.J.); michal.czerwinski@wat.edu.pl (M.C.)

**Keywords:** hydrogen-bond acidic polysiloxane, sensor polymer, coatings for gravimetric sensors, on-site detection of CWAs

## Abstract

In this work, the synthesis of a new polysiloxane, poly {dimethylsiloxane-*co*-[4-(2,3-difluoro-4-hydroxyphenoxy) butyl] methylsiloxane} (dubbed PMFOS), is presented. This polymer exhibits high hydrogen bond acidity and was designed to be used as a sensor layer in gas sensors. The description of the synthetic route of the PMFOS has been divided into two main stages: the synthesis of the functional substituent 4-(but-3-en-1-yloxy)-2,3-difluorophenol, and the post-polymerization functionalization of the polysiloxane chain (methylhydrosiloxane-dimethylsiloxane copolymer) via hydrosilylation. The synthesized material was subjected to instrumental analysis, which confirmed its structure. The performed thermal analysis made it possible to determine some properties important for the sensor application, such as glass transition temperature and decomposition temperature. The results showed that PMFOS meets the requirements for materials intended for use in gas sensors based on acoustoelectric transducers.

## 1. Introduction

Nerve chemical warfare agents (CWAs) are a group of chemical substances classified as weapons of mass destruction. Recently, these substances have been used several times, reminding the public of their military importance. For this reason, it is very important to provide the first responders with the appropriate tools for the fast detection of these substances at the place of their use.

Currently, on-site detection of nerve CWAs is possible with the use of gas analyzers that use various analytical techniques [1,2,3,4]. Among them, techniques allowing for the construction of cheap and compact gas analyzers (e.g., that are possible to be used as on-board equipment for unmanned aerial vehicles) are of particular interest. The above requirements are well met by gravimetric gas sensors based on a variety of acoustoelectric transducers, such as quartz crystal microbalances (QMB) [5], surface acoustic wave (SAW) devices utilizing both Rayleigh and Love waves [6,7], or microcantilever transducers [8,9].

A common design feature of all the above-mentioned devices is the presence of a sensor layer, which is the interface between the analyzed gas and the non-selective transducer part of the sensor. The task of the sensor layer is to selectively interact with the analyte. As a result of this interaction, the mass, elasticity, and electrical properties of the layer change. Changing the layer parameters affects the acoustic wave and, as a result, generates the analytical signal of the sensor. The type and properties of the layer used affect not only the selectivity of the sensor but also its sensitivity and dynamic properties.

One of the most commonly used types of sensor layers is an absorption layer. In this case, the dissolution of the analyte molecules present in the analyzed gas takes place in the volume of the layer. The transport of the analyte continues until a thermodynamic equilibrium is reached. At equilibrium, the partitioning of the analyte between the gas and liquid phases can be described by the value of the partition constant K [10]:

(1) $$K=\frac{C_{S}}{C_{V}}$$

where:

Cs: equilibrium analyte concentration in the sensor layer;
Cv: equilibrium analyte concentration in ambient gas.

In the process of designing and synthesizing materials for sensor layers, the aim is to obtain the greatest possible partition constant while maintaining maximum selectivity.

Absorption-type sensor layers can be made of polymers which are in an elastomeric (rubbery) state at the sensor operating temperature. In this case, the sorption of the analyte to the volume of the sensor layer occurs many times faster than for polymers in the glassy state. This translates into faster sensor operation. Taking into account the fact that the sensor should work at a temperature close to room temperature (the K value usually drops significantly with increasing temperature), and at the same time, that the sensor layer should be in the elastomeric state (to ensure good dynamic properties), it is desirable to use polymers with a low glass transition temperature T_g_. In practice, the T_g_ should be well below room temperature. Due to the natively low T_g_, polysiloxane-based functional materials are of particular interest [11]. In addition, polysiloxanes exhibit several other features desirable in sensor applications, such as chemical and temperature stability or the possibility of immobilization on a quartz substrate.

The solvation properties of functional polymers result from the presence of specific functional groups. Hydrogen-bond acidic (HBA) polysiloxanes usually contain alcoholic or phenolic functional substituents. The positively charged hydrogen atom in the hydroxyl group of such a substituent exhibits a high ability to form a hydrogen bond. This feature is complementary to the relatively specific ability of the nerve CWA molecule to act as a hydrogen acceptor in the hydrogen-bond formation process. Therefore, HBA polysiloxanes are widely used in sensors for the detection of nerve CWA vapors.

**Figure 1:**
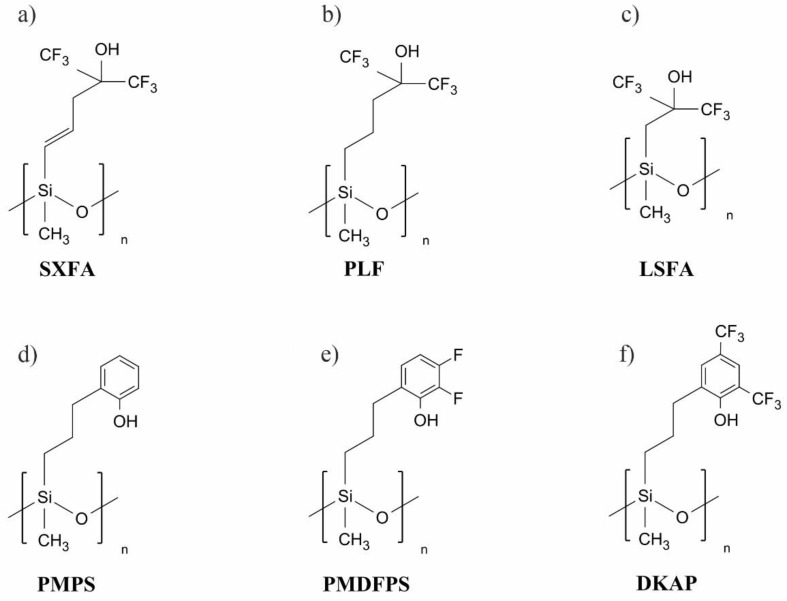
Selected HBA polysiloxanes used in acoustic wave sensors of nerve CWAs and their simulants: (**a**) SXFA [14]; (**b**) PLF [12]; (**c**) LSFA [13]; (**d**) PMPS [17]; (**e**) PMDFPS [18]; (**f**) DKAP [19,20].

A strongly acidic group found in the structure of many HBA polysiloxanes is hexafluoroisopropanol (HFIP) [12,13,14,15,16]. However, it shows a high affinity to water vapor that is always present in the environment. Apart from HFIP-containing polysiloxanes, materials with phenolic substituents, which interact less with water vapor, have also been developed [17,18,19,20]. The structures of selected HBA polysiloxanes used in acoustic wave sensors are shown in Figure 1.

One of the most commonly used methods for the synthesis of functional polysiloxanes is the post-polymerization functionalization of the existing polymethylhydrosiloxane chain via hydrosilylation [13,15,21]. This method has been used many times for the synthesis of the HBA polysiloxanes used in the detection of nerve CWAs and their simulants. The greatest advantage of this method is the possibility of the one-step preparation of functional polysiloxanes.

**Figure 2:**
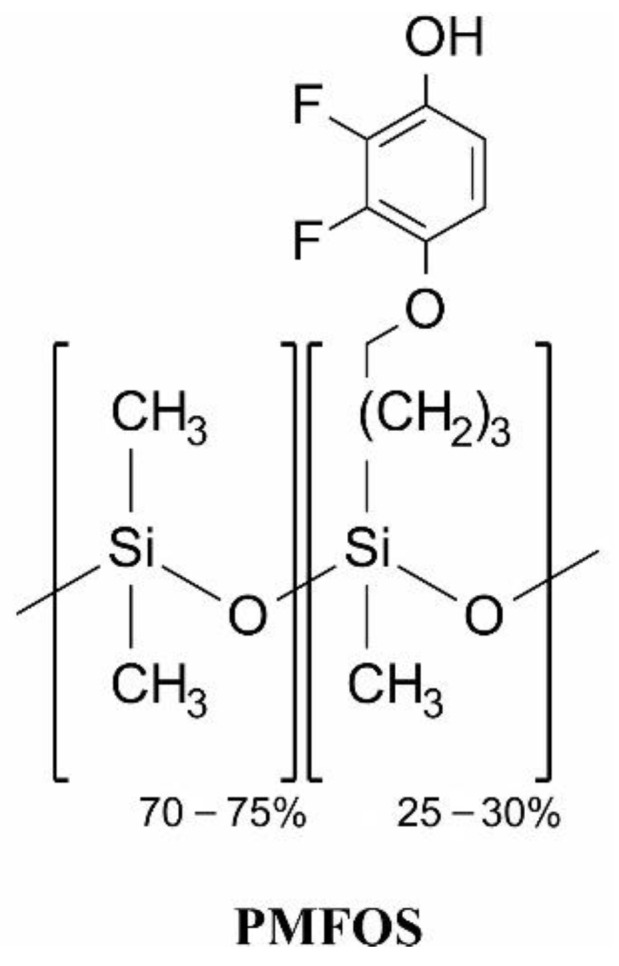
Structure of poly{dimethylsiloxane-*co*-[4-(2,3-difluoro-4-hydroxyphenoxy) butyl] methylsiloxane}—PMFOS.

This work presents the synthesis of a new sensor polysiloxane that contains fluorophenol groups: poly {dimethylsiloxane-*co*-[4-(2,3-difluoro-4-hydroxyphenoxy) butyl] methylsiloxane}, dubbed PMFOS. The structure of PMFOS is presented in Figure 2.

This material is structurally similar to PMDFPS (Figure 1e). An important difference between these materials is the position of the hydroxyl group in the aromatic ring. In the case of PMDFPS, the hydroxyl group is in the ortho position, in relation to the alkyl linker with the polysiloxane chain, which results from the adopted synthetic route using the Claisen rearrangement [18]. In turn, in the case of PMFOS, the -OH group is located in the para position to the alkoxy group, connecting the aromatic ring to the polysiloxane backbone. The transfer of the hydroxyl group to a more exposed position was to minimize steric hindrance during the formation of acidic hydrogen bonds with the analyte molecule. The first step of the PMFOS synthesis was to obtain the functional substituent: 4-(but-3-en-1-yloxy)-2,3-difluorophenol. The functional polymer was then obtained by a post-polymerization functionalization of existing polysiloxanes via Pt-catalyzed hydrosilylation.

PMFOS has already been used in SAW sensors, as is described in our earlier work [22]. Additionally, its solvation properties were determined using inverse gas chromatography, and its acidic nature was confirmed [23].

## 2. Materials and Methods

### 2.1. Materials

All chemicals used in this study were commercially available products. The reagents, 2,3-difluorophenol, but-3-en-1-ol, triphenylphosphine, diisopropyl azodicarboxylate (DIAD), n-butyllithium (*n*-BuLi, a 2.5M solution in hexane), tripropylborate, and potassium peroxymonosulfate (Oxone R), as well as the solvents (tetrahydrofuran-THF, pentane, acetone, toluene) were reagent-grade materials used as supplied by various vendors. As an initial polymer material, a methylhydrosiloxane–dimethylsiloxane copolymer (PMHS) was used (ABCR, Karlsruhe, Germany, 25–30% methylhydrosiloxane–dimethylsiloxane copolymer). A catalyst used in the hydrosilylation reaction was a Karlstedt’s catalyst (ABCR, platinum–divinyltetramethyldisiloxane complex 3–3.5% Pt in vinyl-terminated polysiloxane). The catalyst was further diluted with dry toluene to a concentration of 0.2% Pt.

### 2.2. Instrumentation

The purity and structures of the synthesized substances were confirmed by the use of various analytical methods. The mass spectra were obtained using the gas chromatograph Agilent, Santa Clara, CA, United States, model 6890 N, coupled with a mass spectrometer (GC-MS). Nuclear magnetic resonance (NMR) experiments were conducted using a Bruker, Billerica, MA, United States, model Avance III HD 500 MHz spectrometer (field 11.7 T). The analyses were performed in DMSO with a concentration of 10 wt.%. The 1H and ^13^C spectra were referenced to tetramethylsilane (TMS, δ 0.00 ppm) protons and carbons, respectively. The ^19^F spectra were referenced to CCl_3_F (0.00 ppm). The carbon spectra are protons decoupled with CPD sequence. Studies of infrared (IR) absorption characteristics were carried out using the transmission technique. The Fourier transform infrared (FT-IR) spectrometer used to record spectra was a NICOLETiS10, from Thermo Fisher Scientific, Waltham, MA, United States. Liquid samples of polymers in CCl_4_ were analyzed. The molecular weights and mass distributions of the initial polymer material and synthesized functionalized polysiloxane were determined by gel permeation chromatography (GPC), using the OMNISEC multi-detector GPC/SEC system from Malvern Panalytical, Malvern, United Kingdom. The thermal properties of the synthesized polysiloxane were investigated using a DSC 204F1 Phoenix differential scanning calorimeter (DSC) from Netzsch, Selb, Germany and LabSys thermogravimetric analyzer (TGA), manufactured by the Setaram, Caluire-et-Cuire, France.

### 2.3. Synthesis of Functional Polysiloxane

**Figure 3:**
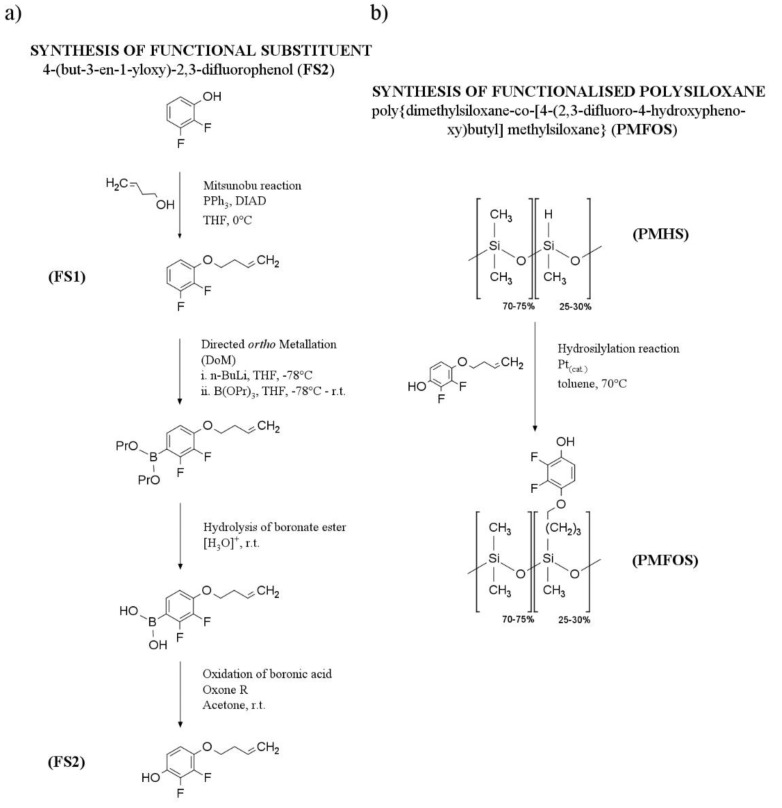
Synthetic protocol of PMFOS.

The synthetic route of PMFOS is presented in Figure 3.

The synthesis of the functional substituent 4-(but-3-en-1-yloxy)-2,3-difluorophenol (FS2) was performed in several successive steps, illustrated in Figure 3a. The first step was to obtain 2,3-difluorophenyl but-3-en-1-yl ether (FS1). FS1 was obtained from 2,3-difluoropheonol and but-3-en-1-ol was obtained via a Mitsunobu reaction. After FS1 was purified by vacuum distillation, the preparation of FS2 was started. In this step, directed ortho metalation (DoM) was used to obtain the arylmetal derivative of FS1. The intermediate product was then trapped with tripropylborate ester. The following hydrolysis of the arylborate ester yielded a boronic acid derivative. The last step was to convert the boronic acid derivative to the target phenol (FS2) by oxidizing the acid with a strong oxidizing agent (Oxone R). FS2, purified by vacuum distillation, was used for further steps in the preparation of PMFOS.

**Figure 4:**
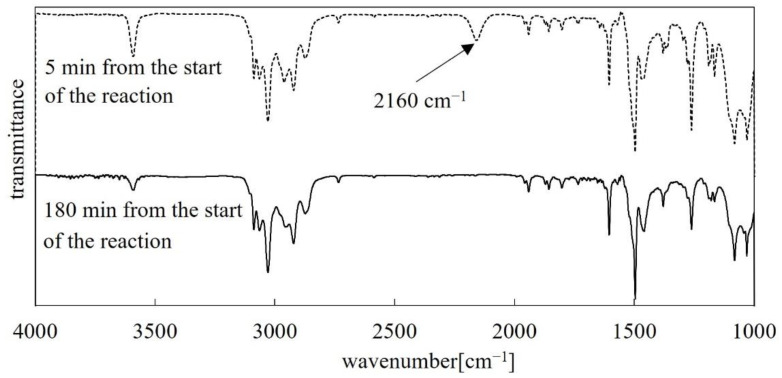
FT-IR spectra of the reaction mixture (FS2, toluene, catalyst, PMHS) recorded: 5 min from the start of the reaction—dotted line; 180 min from the start of the reaction—solid line.

The next step was introducing the functional substituent FS2 into the polysiloxane backbone (PMHS) via a Pt-catalyzed hydrosilylation (Figure 3b). This step of the reaction was carried out under N_2_ with the exclusion of moisture by use of the Schlenk technique. The Pt catalyst used in this reaction was Karstedt’s catalyst (3–3.5% Pt). The progress of the reaction was monitored by FT-IR to observe the disappearance of the Si–H stretching band at 2157.6 cm^−1^ (Figure 4).

After the complete conversion of the Si−H bonds indicated by FT-IR, the reaction was stopped. The reaction mixture was then filtered and the solvent was removed by rotary evaporation to yield the desired PMFOS as a dense, light grey oil. A detailed recipe for the synthesis of the FS2 and PMFOS, as well as the results of the MS, FT-IR, and NMR analyses, are included in Supplementary Material.

## 3. Results and Discussion

The synthesized PMFOS was subjected to FT-IR, NMR, GPC, DSC, and TG analysis.

### 3.1. FT-IR Analysis

**Figure 5:**
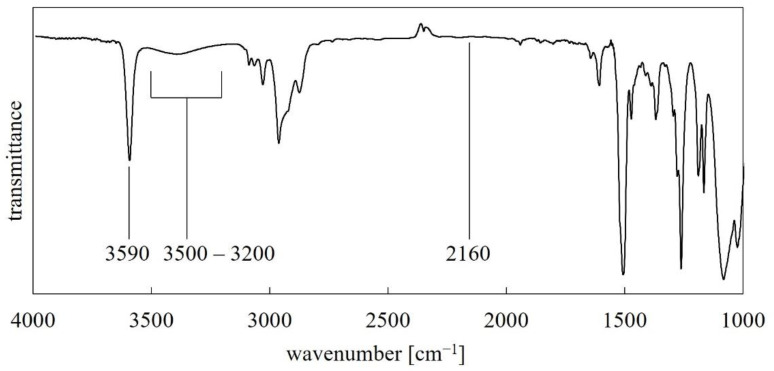
FT-IR spectrum of PMFOS.

The obtained PMFOS infrared spectrum is shown in Figure 5.

The absence of an absorption peak of about 2160 cm^−1^ indicates that the hydrosilylation was completed. The sharp, strong absorption band from free hydroxyl groups at 3590 cm^−1^ is noteworthy. In the range of 3500–3200 cm^−1^, a broad band of low intensity is also observed, which can be attributed to the hydroxyl groups involved in hydrogen bonding (even though the material was vacuum dried before the IR analysis).

### 3.2. NMR Analysis

**Figure 6:**
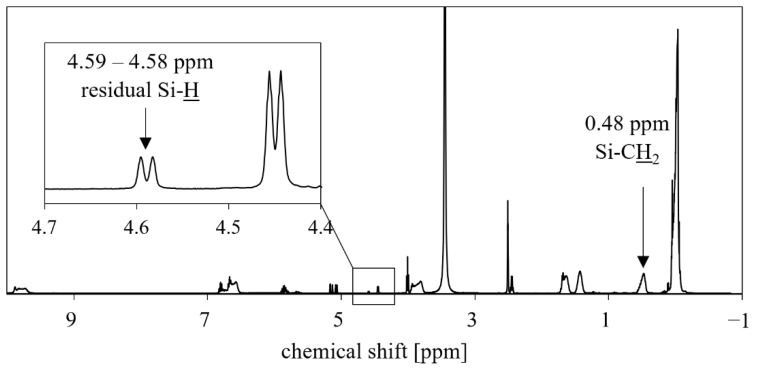
^1^H-NMR spectrum of PMFOS.

PMFOS was analyzed by ^1^H-NMR, ^13^C-NMR, and ^19^F-NMR. The ^1^H-NMR spectrum is shown in Figure 6.

The NMR analysis confirmed the presence of the expected structural formations in the PMFOS. In the above ^1^H-NMR spectrum, a small peak in the range of chemical shifts corresponding to ^1^H in the Si−H bond (4.59–4.58 ppm) is observed. This is indicative of a residual Si−H bond from the initial PMHS that was not converted. The Si−H band was not visible in the IR spectrum, however. This is probably due to the differences in the sensitivities of the two methods, or the particular conditions of the analyses. Based on the ^1^H-NMR PMFOS and PMHS spectra, as well as the knowledge of the initial content of the Si−H bonds declared by the PMHS manufacturer, the conversion rate of the Si−H bond in the PMFOS material was estimated. Thus, the determined conversion rate was 97.2% (see Supplementary Material). The above spectrum also shows the peak corresponding to the protons in Si−CH_2_ (0.48 ppm). The presence of this peak indicates the attachment of the carbon chain of the functional substituent to the silicon atom.

### 3.3. GPC Analysis

**Figure 7:**
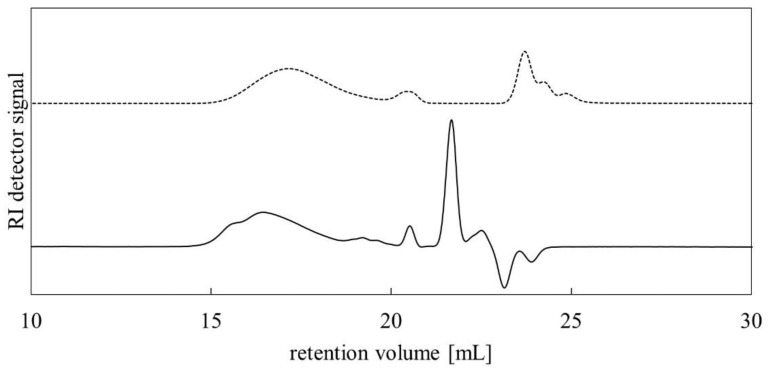
GPC chromatograms of initial PMHS (dotted line) and synthesized PMFOS (solid line).

The synthesized PMFOS and the initial PMHS were subjected to GPC analysis. Figure 7 shows GPC chromatograms of PMFOS and PMHS materials recorded with a refractive index (RI) detector.

Based on the determined molecular weight distributions of PMFOS and PMHS, the number-average molecular weights (M_n_), the mass-average molecular weights (M_w_), and the polydispersities (Đ) of both polymers were determined. In the case of PMFOS, M_n_ = 9800 g/mol and Đ = 2.92 was obtained, while for PMHS, it was M_n_ = 3400 g/mol and Đ = 2.25. Comparing the obtained M_n_ value, it can be said that, in the course of the reaction, the average molecular weight of the PMHS substrate increased. The obtained weight gain corresponds to the introduction of functional substituent molecules into the polysiloxane backbone in an amount close to the content of the Si−H bonds declared by the PMHS manufacturer.

### 3.4. Thermal Analysis

**Figure 8:**
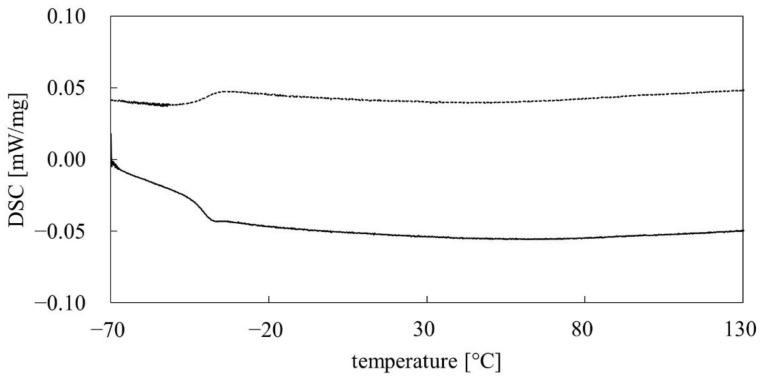
DSC thermograms of PMFOS (cooling curve—dotted line; heating curve—solid line).

The phase transition analysis was performed under an inert gas atmosphere. The polymer was first heated from −70 °C to 130 °C and then cooled back to −70 °C. The obtained PMFOS thermograms are shown in Figure 8.

Based on the performed DSC analysis, the glass transition temperature T_g_ of the PMFOS material was determined. The T_g_ determined for the heating and cooling of the sample have convergent values of −44.0 and −44.3 °C, respectively. A DSC analysis of the initial PMHS was also performed. In this case, no phase transitions in the temperature range −70 to 130 °C were observed. On this basis, the T_g_ for PMHS was found to be below −70 °C. As expected, the introduction of the fluorophenol functional substituent increased the glass transition temperature of PMFOS relative to the initial PMHS. Nevertheless, the T_g_ of PMFOS is still relatively low. A comparison of the T_g_ values of selected HBA polymers is presented in Table 1.

By analyzing the values from Table 1, it can be concluded that the T_g_ of PMFOS is much lower than for most materials of this type. In this comparison, only the SXFA material has an equally low T_g_.

**Figure 9:**
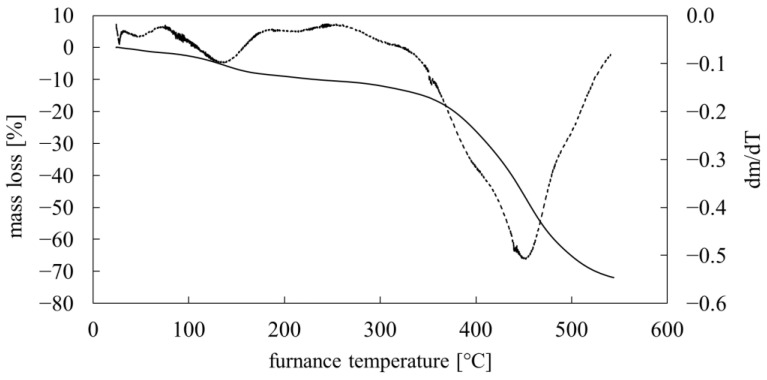
TG curve of PMFOS (mass loss versus temperature— solid line, first derivative of mass loss versus temperature— dotted line).

A TG analysis of the PMFOS was also performed. The TG thermogram is shown in Figure 9. The analysis was performed under an inert gas atmosphere (argon).

A 5% decrease in the sample mass was recorded at a temperature of about 131 °C, and a 10% decrease was recorded at a temperature of about 232 °C. From about 280 °C, the PMFOS began to rapidly degrade. Part of the weight loss in the initial part of the thermogram (approx. 1.69%, up to approx. 75 °C) can be attributed to the loss of moisture and residual organic solvents.

Summarizing the conducted thermal analysis, it can be stated that PMFOS is characterized by a suitably low T_g_ for sensor applications. In addition, the material exhibits good thermal stability, even at temperatures much higher than the typical operating conditions of gas sensors with absorption layers.

### 3.5. Application in SAW Sensors

PMFOS was used as a sensor layer for gas sensors based on 195 MHz dual-port SAW resonators [28], as shown in our recent paper [22]. In the process of sensor preparation, PMFOS layers ware applied onto SAW resonators using thermal evaporation. These studies have shown that PMFOS sensors are highly selective to hydrogen-bond bases, which include nerve CWAs and their simulants such as dimethyl methylphosphonate (DMMP) or triethyl phosphate (TEP).

**Figure 10:**
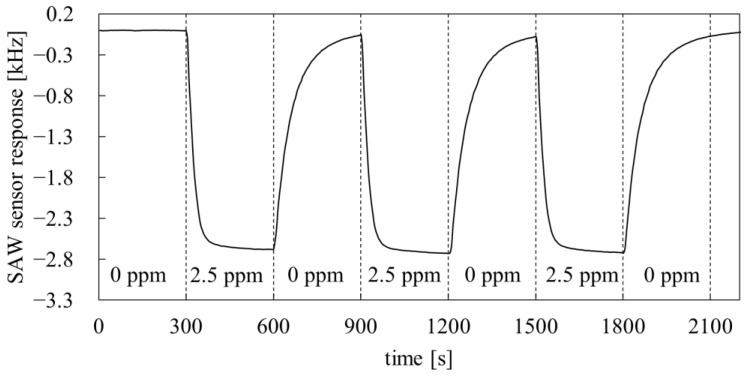
The real-time PMFOS sensor response recorded during alternating exposure to a gas stream with a DMMP concentration of 2.5 ppm and dry synthetic air at 30 °C.

Selectivity and sensitivity are not the only parameters that characterize the suitability of the sensor. For the detection of nerve CWAs, a short response time is also important. In order to investigate the dynamic properties of PMFOS sensors, which are highly influenced by the PMFOS T_g_, additional measurements were made. The response time of PMFOS sensors to DMMP vapor was investigated at 30 °C, which is well above the T_g_ of PMFOS (−44 °C). The temperature of 30 °C is close to room temperature, and providing sensor thermostating at such a temperature is not problematic, which is particularly important in the case of miniature, portable devices. The real-time DMMP response curve of the PMFOS sensor is presented in Figure 10.

As shown in the Figure 10, the sensor responds rapidly to the changes in the analyte concentration. In the case of DMMP, the value of the response time constant is 14.6 +/− 0.9 s. Such a low value of the response time constant results mainly from the high vapor diffusion rate in the volume of the sensor layer. As the results show, the PMFOS sensor can be used at temperatures close to room temperature while maintaining good dynamic properties.

## 4. Conclusions

This paper presents the synthesis of a new sensor polysiloxane that can be used in SAW sensors. The synthesized material was characterized by several instrumental methods that confirmed its structure and made it possible to determine some thermal properties important for sensor applications. Summarizing the research performed, we can say:

- the developed synthetic route was well established and verified with instrumental methods;
- the synthetic route allows for obtaining the product with high yield (functional substituent step yield: 74%, hydrosilylation step yield: 88%);
- the determined thermal properties of PMFOS (T_g_ and decomposition temperature) meet the requirements for materials intended for use in gas sensors based on acoustoelectric transducers;
- the research on the dynamic properties of the PMFOS sensors has shown that they are characterized by short response time to DMMP vapor (several seconds). The response time at this level is appropriate for the detection of highly toxic substances.

## Figures and Tables

**Table 1:** Comparison of glass transition temperatures (T_g_) of selected HBA polymers used in gas sensors.

Polymer	T_g_ (°C)	Reference
PMFOS	−44	This work
SXFA	−44	[24]
PLF	−17	[25]
BSP-3 ^1^	6	[26]
DKAP	20	[27]

^1^ The acronym BSP-3 stands for a hybrid organic/inorganic polymer incorporating fluoroalkyl-substituted bisphenol groups linked using oligosiloxane spacers, reported in work [26]. The structures of the remaining polymers are shown in Figure 1.

## Data Availability

The data presented in this study are available on request from the corresponding author.

## Supplementary Materials

The following supporting information can be downloaded at: https://www.mdpi.com/article/10.3390/polym14061147/s1, Figure S1: Synthetic protocol of PMFOS; Figure S2: Mass spectrum of FS1; Figure S3: Mass spectrum of FS2; Figure S4: Mass spectrum of FS2; Figure S5: ^1^H NMR spectrum of 4-(but-3-en-1-yloxy)-2,3-difluorophenol (FS2); Figure S6: 1H NMR spectrum of 4-(but-3-en-1-yloxy)-2,3-difluorophenol (FS2); Figure S7: The section of FS2 ^13^C-NMR spectrum from 139.5 to 143.0 ppm; Figure S8: FT-IR spectrum of poly{dimethylsiloxane-co-[4-(2,3-difluoro-4-hydroxyphenoxy) butyl] methylsiloxane} – PMFOS; Figure S9: ^1^H-NMR spectrum of PMFOS; Figure S10 : ^13^C-NMR spectrum of PMFOS; Figure S11: ^19^F-NMR spectrum of PMFOS; Figure S12: ^1^H-NMR spectrum of PMHS with integrated peaks of Si-H (4.61–4.69 ppm) and Si-CH3 (−0.10–0.05 ppm); Figure S13: ^1^H-NMR spectrum of PMFOS with integrated peaks of Si-H (4.57–4.61 ppm) and Si-CH3 (−0.10–0.05 ppm); Supporting information for Fluorophenol-containing hydrogen bond acidic polysiloxane for gas sensing—synthesis and characterization.
