# Supplementary material for: Fluorophenol-Containing Hydrogen-Bond Acidic Polysiloxane for Gas Sensing-Synthesis and Characterization
Source: Polymers (Basel). 2022 Mar 13;14(6):1147. doi: 10.3390/polym14061147 (PMC8949868; doi:10.3390/polym14061147)
Supplement: Supplementary file 1 [file polymers-14-01147-s001.zip › polymers-1621594-supplementary.pdf]

Supporting information for

Fluorophenol-containing hydrogen bond acidic polysiloxane for gas sensing - synthesis and characterization

Michał Grabka[a,*], Przemysław Kula[a], Mateusz Szala[a], Krzysztof Jasek[a], Michał Czerwiński[a]
[a]Institute of Chemistry, Military University of Technology, Warsaw 00-908, Poland

*Corresponding author:
e-mail address: michal.grabka@wat.edu.pl
postal address: gen. Sylwestra Kaliskiego 2 street, 00-908 Warsaw, Poland

Contents:

1. Synthesis recipe of poly{dimethylsiloxane-co-[4-(2,3-difluoro-4-hydroxyphenoxy) butyl] methylsiloxane} – PMFOS;
2. Instrumental analysis of intermediates and products
   a) Mass spectrum of but-3-en-1-yl 2,3-difluorophenyl ether (FS1);
   b) Mass spectrum of 4-(but-3-en-1-yloxy)-2,3-difluorophenol (FS2);
   c) FT-IR spectrum of 4-(but-3-en-1-yloxy)-2,3-difluorophenol (FS2);
   d) ^{1}H and ^{13}C-NMR investigation of 4-(but-3-en-1-yloxy)-2,3-difluorophenol (FS2);
   e) FT-IR spectrum of poly{dimethylsiloxane-co-[4-(2,3-difluoro-4-hydroxyphenoxy) butyl] methylsiloxane} – PMFOS;
   f) ^{1}H, ^{13}C and ^{19}F-NMR investigation of poly{dimethylsiloxane-co-[4-(2,3-difluoro-4-hydroxyphenoxy) butyl] methylsiloxane} – PMFOS;
   g) Calculation of the conversion rate of Si-H group.

1. **Synthesis recipe of poly{dimethylsiloxane-co-[4-(2,3-difluoro-4-hydroxyphenoxy) butyl] methylsiloxane} – PMFOS**

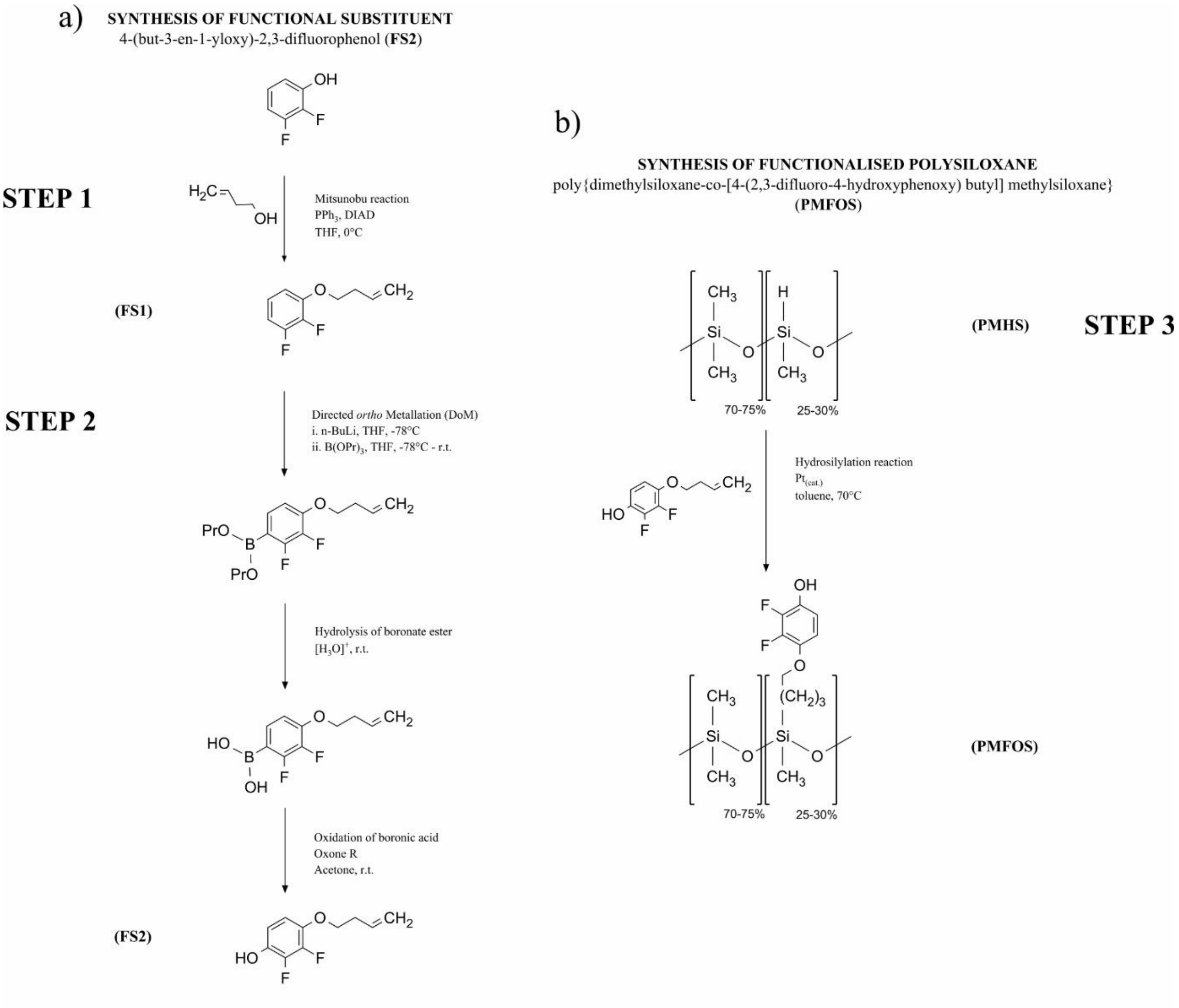

Figure S1. Synthetic protocol of PMFOS.

RECIPE:

Step 1. Synthesis of but-3-en-1-yl 2,3-difluorophenyl ether (FS1) via a Mitsunobu reaction.

2,3-difluorophenol (0.15 mol), but-3-en-1-ol (0.18 mol), triphenylphosphine (0.21 mol) were dissolved in dry THF (150 cm^3) under N_2 atmosphere. Then the mixture was cooled down to 0 °C. Afterward, DIAD (0.21 mol) was added slowly dropwise. The temperature of 0 °C was maintained during the addition of DIAD. After that, mixture was left to reach room temperature (r. t.) with stirring yet for 24 h. The crude reaction mixture was filtered, washed with pentane and concentrated to dryness by rotary evaporation. Residue was distilled under reduced pressure to give desired intermediate product FS1. FS1 was clear, colorless liquid (0.14 mol, 93.3 % yield, MS, m/z: 184 (M^+)).

Step 2. Synthesis of 4-(but-3-en-1-yloxy)-2,3-difluorophenol (FS2) via directed ortho metalation (DoM)

FS1 (0.10 mol) was dissolved in dry THF (100 cm^3). The mixture was cooled down to -78 °C under N_2 atmosphere. Then the n-BuLi (2.5 M in hexane, 44 cm^3) was added carefully

dropwise. During the addition of the n-BuLi solution, the mixture was vigorously stirred. The rate of dropping was constantly monitored in order to not exceed the mixture temperature of -70 °C (forming of the arylmetal intermediate is exothermic). After completion of n-BuLi addition, the temperature of -78 °C was maintained for a further 2 h. Afterward, tripropyl borate (0.12 mol, 26.3 cm^3) was added slowly dropwise. As before, during the addition of tripropyl borate mixture was vigorously stirred and the temperature below -70 °C was maintained. The reaction mixture was then left to reach r. t. Excess of solvent from the crude reaction mixture was removed by rotary evaporation. Distilled water (200 cm^3) with the addition of HCl was added to the residue in order to hydrolyze formed boronate ester. After a few minutes, a light brown precipitate of boronic acid formed. Boronic acid was then separated from the reaction mixture by vacuum filtration, washed with pentane and dried. Finally, 0.06 mol (59.8 % yield) of a crystalline white boronic acid was collected. In order to obtain FS2 boronic acid was oxidized. Boronic acid (0.03 mol) was dissolved in acetone. Then the solution of Oxone R (0.2 M in water, 180 cm^3) was added. The reaction mixture was stirred for 10 minutes in r. t. The FS2 was extracted from the crude reaction mixture with pentane. After removing the solvent by rotary evaporation the residues (dense, yellowish oil) were distilled under reduced pressure to give desired crystalline white FS2 (0.024 mol, 80.0 % yield; MS: m/z: 200(M^+)).

Step 3. Introducing the FS2 into polysiloxane backbone (PMHS) via hydrosilylation.
This step of the reaction was carried out under N_2 with the exclusion of moisture by use of Schlenk technique. To the mixture of FS2 (0.007 mol) and Karstedt's catalyst (0.14 g, 0.2 % Pt) in dry toluene (10 cm^3) the PMHS (1.5 g, 0.007 mol of Si-H – the amount of the polymer was calculated on the basis of the average content of monomers with Si-H bond adopted on the basis of the PMHS manufacturer's declaration as 27.5%) was added slowly dropwise. The mixture was refluxed for 6 h in 70 ˚C under N_2. The progress of the reaction was monitored by FT-IR to observe the disappearance of the Si-H stretching band at 2157.6 cm^{-1}. After complete disappearance of this band mixture was cooled to r. t. The mixture was then filtered under vacuum and the solvents removed by rotary evaporation. Finally, grey liquid poly{dimethylsiloxane-co-[4-(2,3-difluoro-4-hydroxyphenoxy) butyl] methylsiloxane} - PMFOS was collected (2.54 g, yield: 88%).

2. **Instrumental analysis of intermediates and products**

a) Mass spectrum of but-3-en-1-yl 2,3-difluorophenyl ether (FS1).

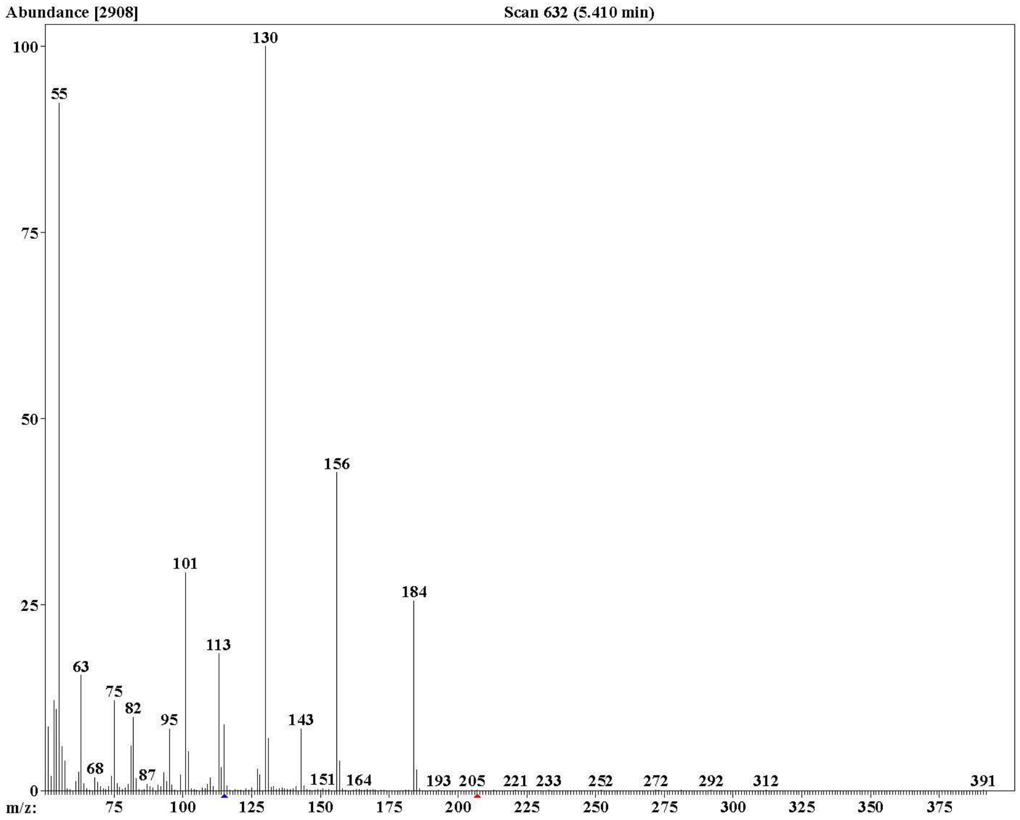

Figure S2. Mass spectrum of FS1.

b) Mass spectrum of 4-(but-3-en-1-yloxy)-2,3-difluorophenol (FS2).

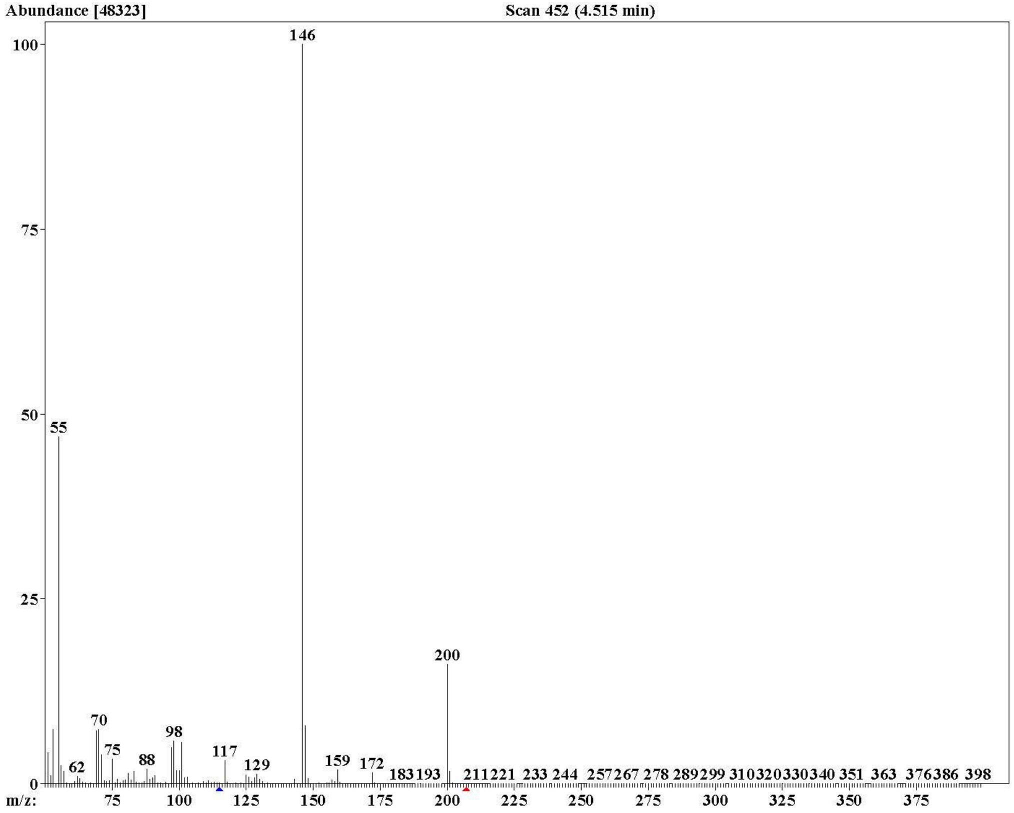

Figure S3. Mass spectrum of FS2.

c) FT-IR spectrum of 4-(but-3-en-1-yloxy)-2,3-difluorophenol (FS2).

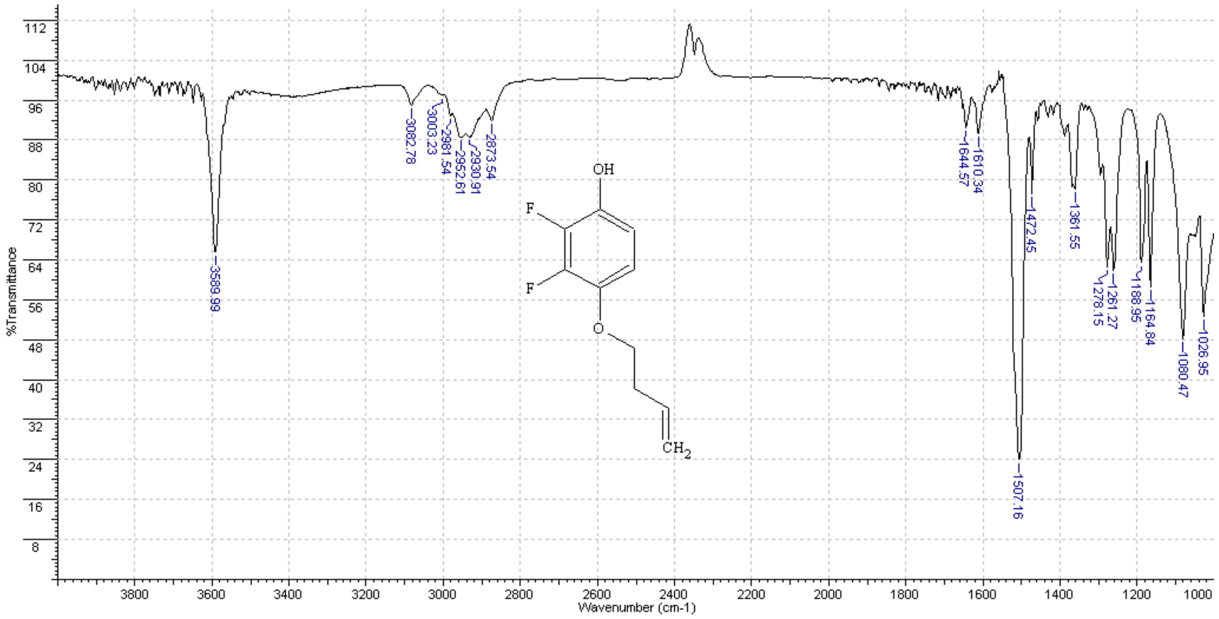

Figure S4. FT-IR spectrum of FS2.

FT-IR[cm^{-1}]: O-H stretch in free hydroxyl groups at 3589.99; Ar-H stretches from 3082.78 to 3003.23; aliphatic C-H symmetrical and asymmetrical stretches from 2981.54 to 2873.54; alkene C=C stretch at 1644.57; aromatic C-C stretches at 1610.34; 1507.16 and 1472.45; Ar-O-H bending at 1361.55; Ar-O-C asymmetrical stretch at 1278.15; Ar-O-H asymmetrical stretch at 1261.27; Ar-F stretch at 1188.95; aliphatic C-C stretch at 1164.84; Ar-O-C symmetrical stretch at 1080.40; Ar-O-H symmetrical stretch at 1026.95.

d) ^{1}H and ^{13}C-NMR investigation of 4-(but-3-en-1-yloxy)-2,3-difluorophenol (FS2).

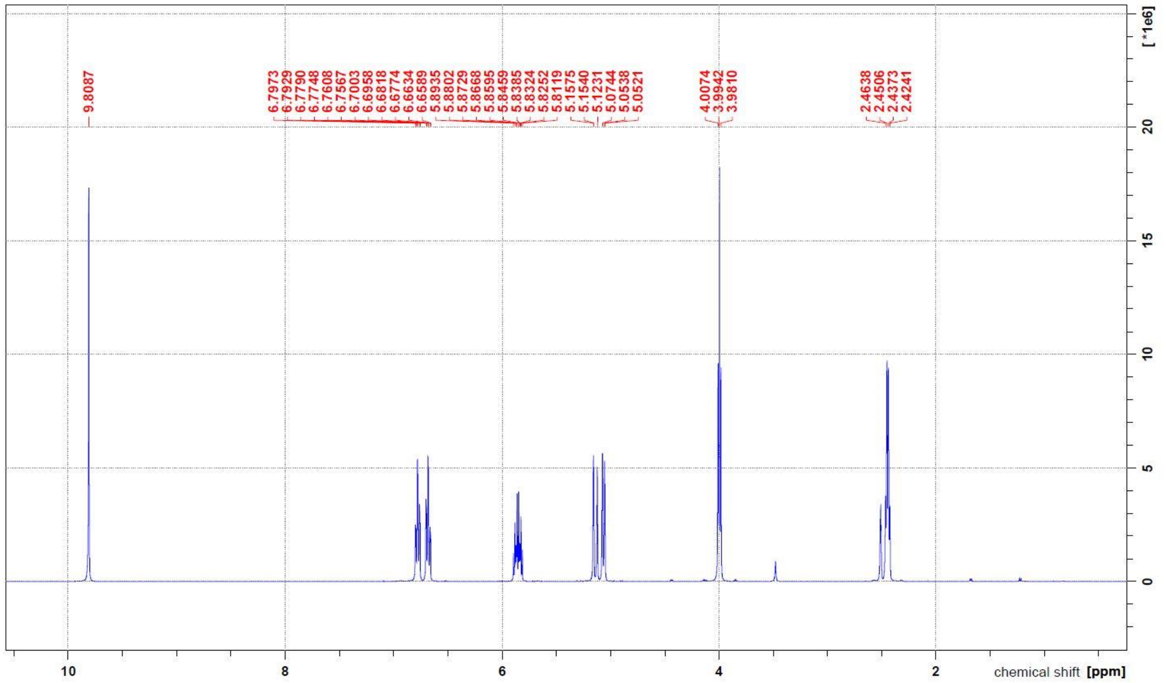

Figure S5. ^{1}H NMR spectrum of 4-(but-3-en-1-yloxy)-2,3-difluorophenol (FS2).

^{1}H NMR[ppm]: 2.45 (q, 2H, CH_2); 3.99 (t, 2H, CH_2); 5.10 (dd, 2H, $=CH_2$); 5.85 (m, 10 lines, 1H, =CH); 6.69 (dt, 2H, $C_{Ar}H$); 9.81 (s, 1H, OH).

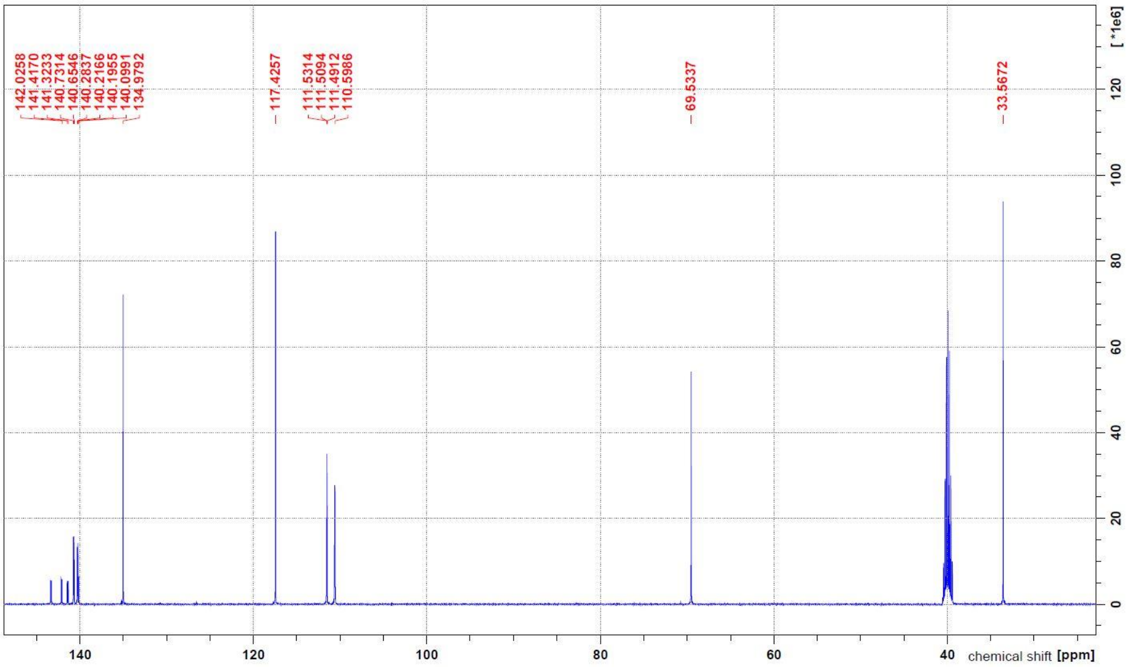

Figure S6. ^{13}C NMR spectrum of 4-(but-3-en-1-yloxy)-2,3-difluorophenol (FS2).

^{13}C {^{1}H} NMR[ppm]: 33.6 (s, CH_2); 69.5 (s, O-CH_2); 110.6 (s, C_{Ar}-H); 111.5 (s, C_{Ar}-H); 117.4 (s, $=CH_2$); 134.9 (s, =CH); 140.1 (d, $^2J_{CF}$=11.8 Hz , C-OH); 140.2 (d, $^3J_{CF}$=8.4 Hz, C_{Ar}(3)-F); 140.6 (d, $^3J_{CF}$=9.5 Hz, C_{Ar}(3)-F); 141.3 (d, $^1J_{CF}$=244.1 Hz, C_{Ar}(2)-F); 142.0 (d, $^2J_{CF}$=11.81 Hz, C_{Ar}-O-); 143.3 (d, $^1J_{CF}$=244.1 Hz, C_{Ar}(3)-F).

In the case of directed ortho metalation of FS1, there was a probability of 2-(but-3-en-1-yloxy)-3,4-difluorophenol formation instead of 4-(but-3-en-1-yloxy)-2,3-difluorophenol. However, a deeper analysis of the product C-NMR spectrum, with detectable C-F couplings, confirmed that the resulting compound was FS2.

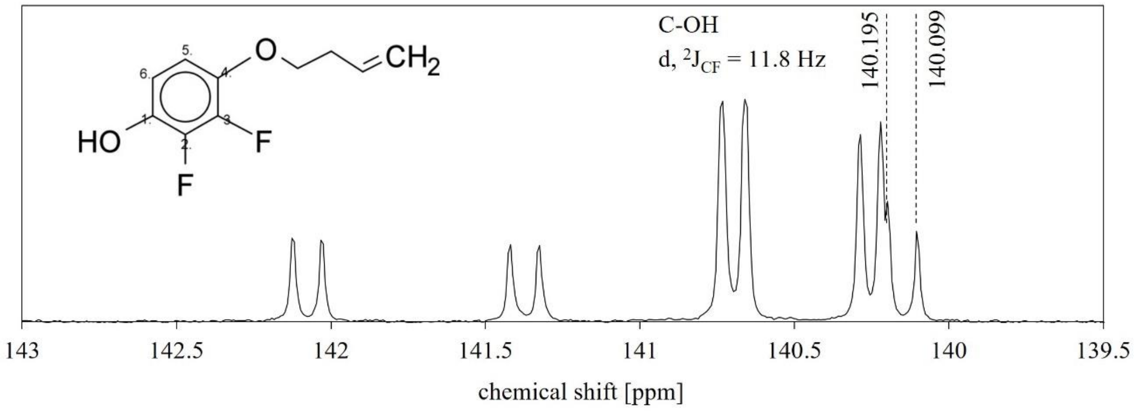

Figure S7. The section of FS2 ^{13}C-NMR spectrum from 139.5 to 143.0 ppm.

In the Figure S7, which is a section of the ^{13}C-NMR spectrum (Figure S6), the doublet that can be assigned to carbon no. 1 attached directly to the hydroxyl group is indicated (dotted lines). The coupling constant between the carbon and fluorine nuclei is 11.8 Hz. Such a high value of the coupling constant indicates the proximity of the fluorine nucleus, which proves the presence of the hydroxyl group at the carbon atom no. 1. If the hydroxyl group was located at the carbon atom no. 5, the C-OH carbon would be separated from the nearest fluorine nucleus by three bonds. In that case, the coupling constant would be much lower and a single peak at about 140.2 ppm would be observed in the spectrum.

e) FT-IR spectrum of poly{dimethylsiloxane-co-[4-(2,3-difluoro-4-hydroxyphenoxy) butyl] methylsiloxane} – PMFOS.

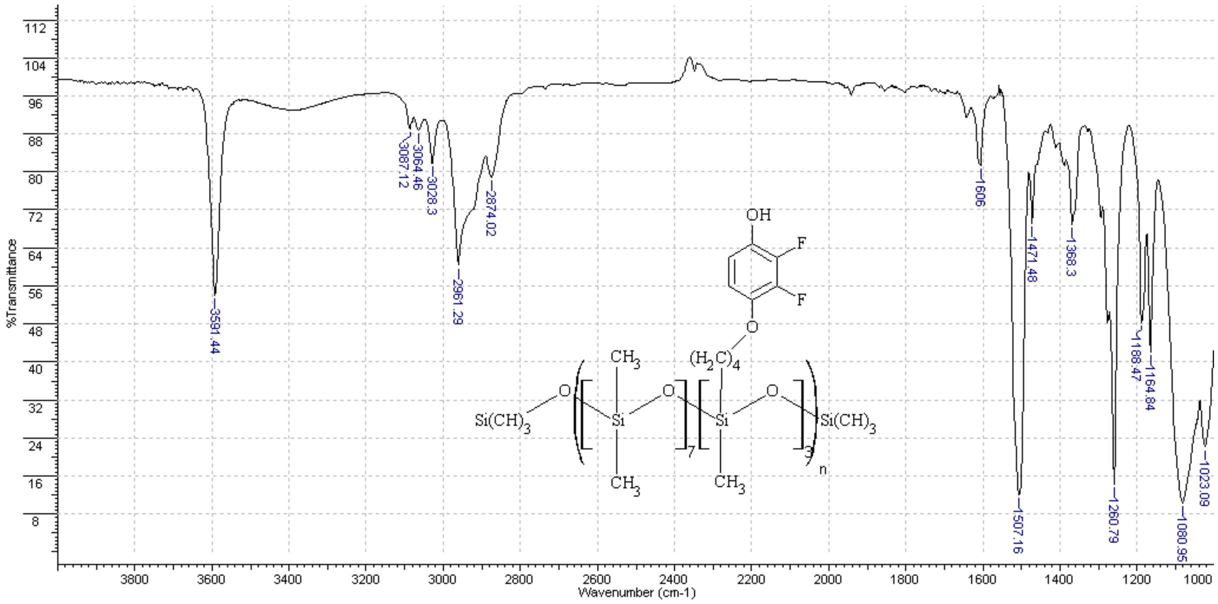

Figure S8. FT-IR spectrum of poly{dimethylsiloxane-co-[4-(2,3-difluoro-4-hydroxyphenoxy) butyl] methylsiloxane} – PMFOS.

FT-IR[cm^{-1}]: O-H stretch in free hydroxyl groups at 3591.44; Ar-H stretches from 3087.12 to 3028.30; aliphatic C-H symmetrical and asymmetrical stretches from 2961.29 to 2874.02; aromatic C-C stretches at 1606.00; 1507.16 and 1471.48; Ar-O-H bending at 1368.30; Si-C stretch at 1260.79; Ar-F stretch at 1188.47; aliphatic C-C stretch at 1164.84; Si-O stretches in linear polysiloxane from 1080.95 to 1023.09. Probably peaks of Ar-O-C and Ar-O-H asymmetrical stretches are covered by the strong peak of Si-C at 1260.79 as well as peaks of Ar-O-C and Ar-O-H symmetrical stretches are covered by deep absorption peak of linear polysiloxane Si-O bonds at 1080.95-1023.09.

f) ^{1}H, ^{13}C and ^{19}F-NMR investigation of poly{dimethylsiloxane-co-[4-(2,3-difluoro-4-hydroxyphenoxy) butyl] methylsiloxane} – PMFOS.

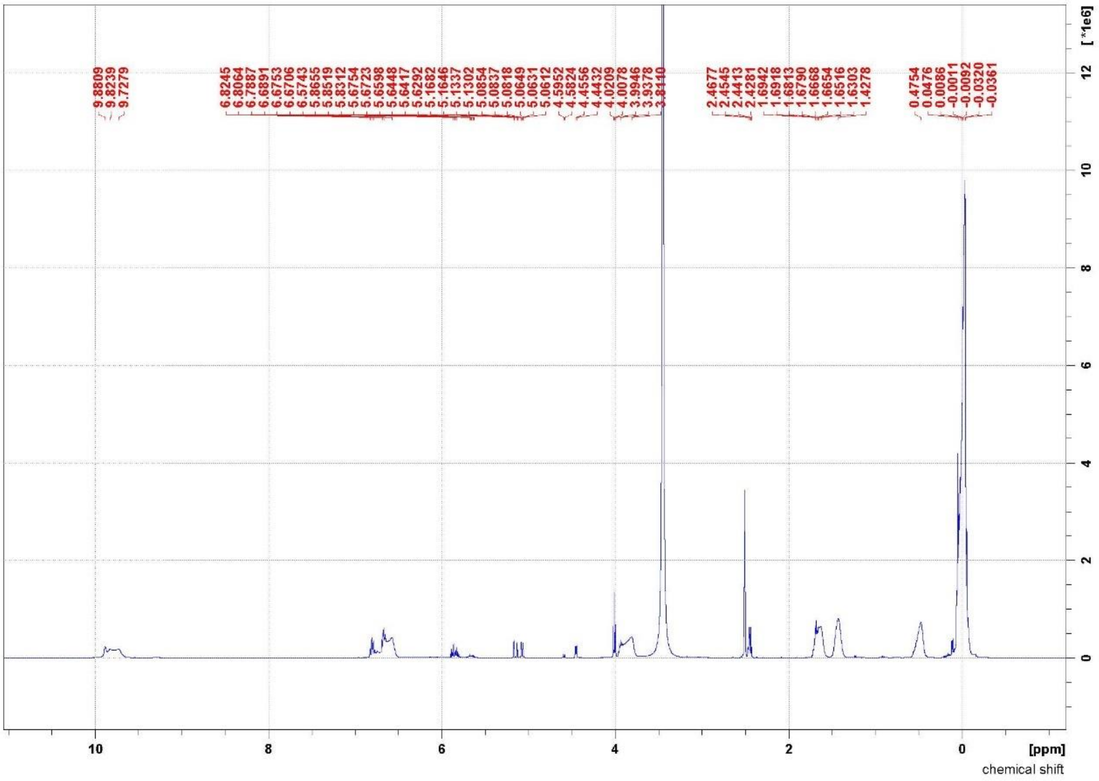

Figure S9. ^{1}H-NMR spectrum of PMFOS.

^{1}H-NMR (500 MHz, DMSO)[ppm]: -0.10 - 0.05 (m, Si-CH_3); 0.4 (m, Si-CH_2); 1.6 (m, CH_2) 2.45 (m, CH_2); 3.8 - 4.0 (m, CH_2); 5.11 (m, =CH_2); 5.9 (m, =CH); 6.7 (m, $C_{Ar}H$); 9.7 - 9.9 (m, OH).

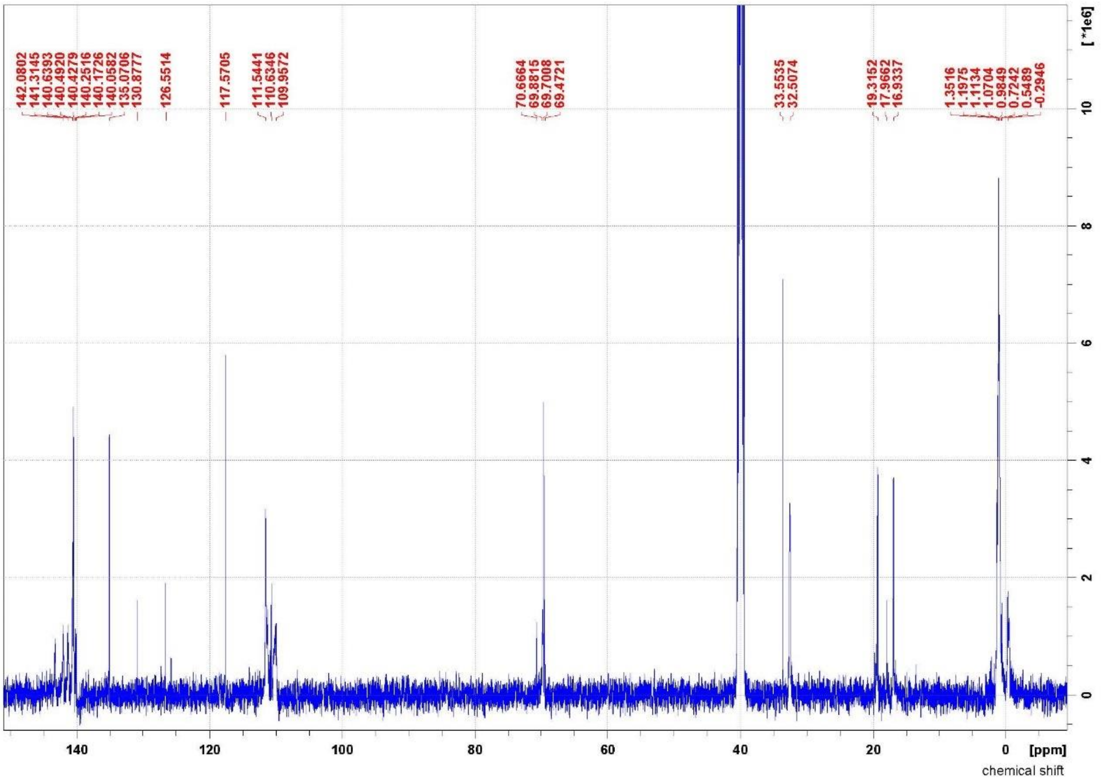

Figure S10. ^{13}C-NMR spectrum of PMFOS.

^{13}C {^{1}H} NMR (500 MHz, DMSO)[ppm]: 1.1 (m, Si-CH_3); 16.9 (s, O-CH_2); 19.3 (s, O-CH_2); 33.6 (s, CH_2); 69.8 (m, O-CH_2); 110.6 (m, C_{Ar}-H); 111.5 (s, C_{Ar}-H); 117.6 (s, =CH_2); 135.1 (s, =CH); 140.3 (d, $^{2}J_{CF}$=11.8 Hz , C-OH); 140.4 (d, $^{3}J_{CF}$=8.4 Hz, C_{Ar}(3)-F); 140.5 (d, $^{3}J_{CF}$=9.5 Hz, C_{Ar}(3)-F); 140.6 (d, $^{1}J_{CF}$=244.1 Hz, C_{Ar}(2)-F); 141.3 (d, $^{2}J_{CF}$=11.81 Hz, C_{Ar}-O-); 142.1 (d, $^{1}J_{CF}$=244.1 Hz, C_{Ar}(3)-F).

The ^{13}C-NMR spectrum shows peaks at 117.6 (s, =CH_2) and 135.1 (s, =CH). This indicates the presence of unreacted, residual FS2. The reason for incomplete conversion of FS2 is that the amount of FS2 added to reaction mixture was calculated on the basis of the average content of monomers with Si-H bond in PMHS (27,5%). Perhaps the actual content of monomers with Si-H was less than 27,5% and therefore residual, unreacted FS2 contaminated the product. The amount of unreacted FS2 has not been determined but due to the fact that FS2 is not visible in the FT-IR spectrum of PMFOS (Figure S8) its amount is probably insignificant.

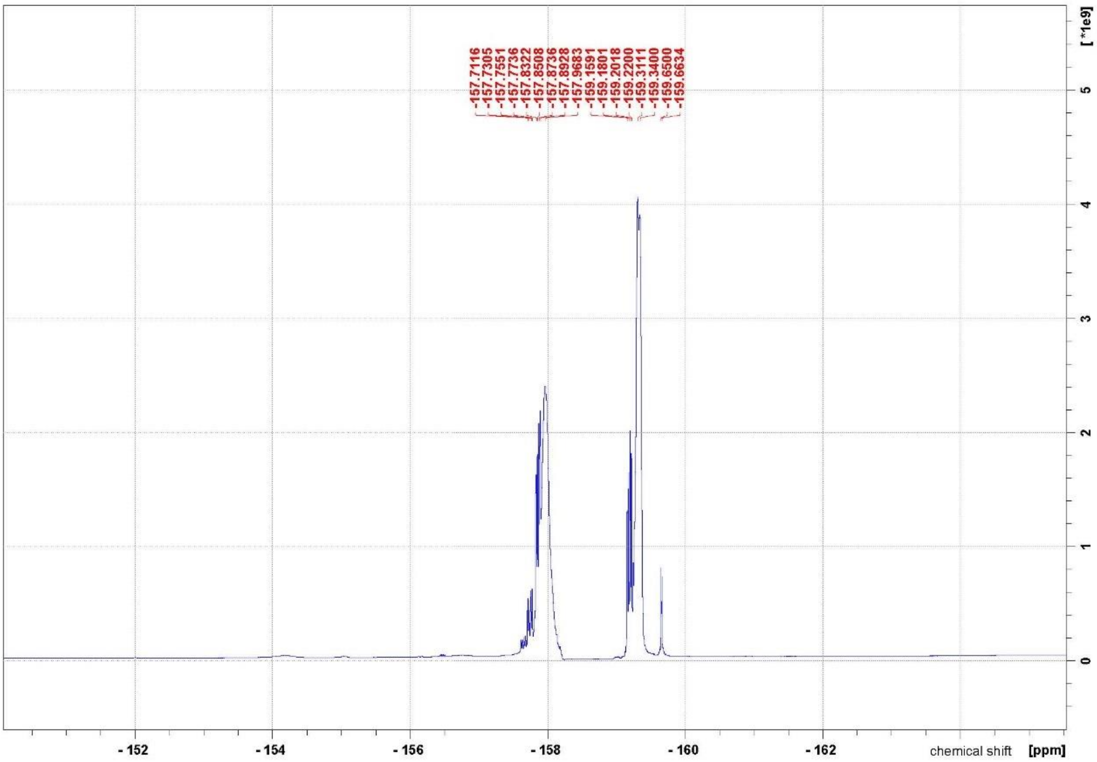

Figure S11. ^{19}F-NMR spectrum of PMFOS.

^{19}F-NMR (500 MHz, DMSO) [ppm]: -159.3 (m, C2-F); -157.8 (m, C3-F).

g) Calculation of the conversion rate of Si-H group.

The ^{1}H-NMR spectra of PMHS in DMSO (Figure S12) and PMFOS in DMSO (Figure S13) were used to calculate the conversion rate of Si-H group.

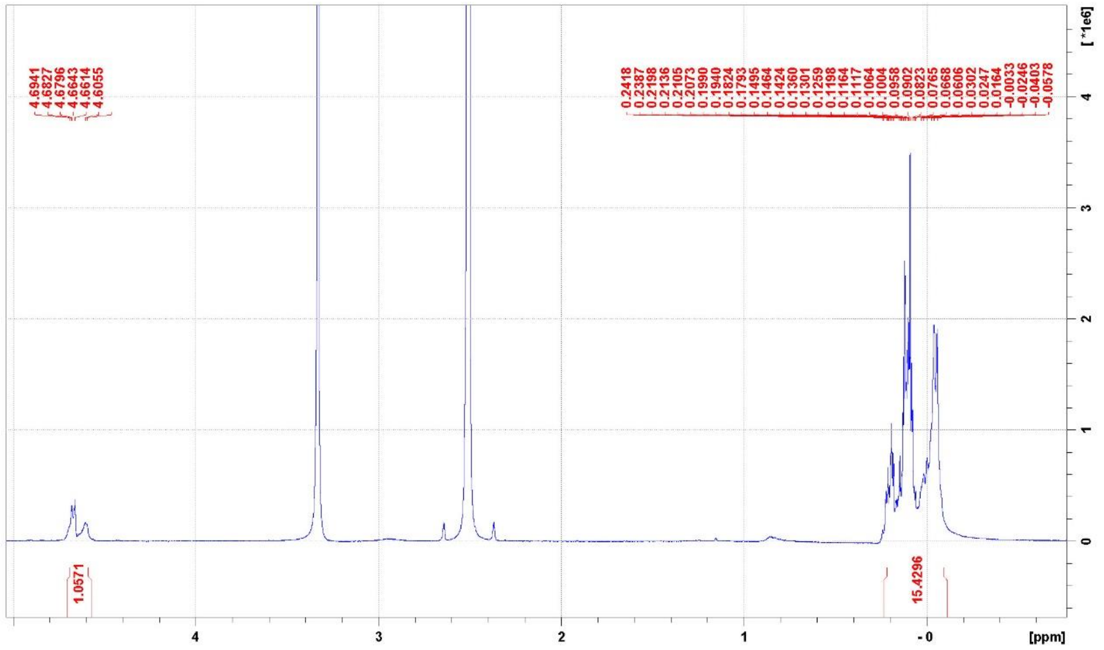

Figure S12. ^{1}H-NMR spectrum of PMHS with integrated peaks of Si-H (4.61 - 4.69 ppm) and Si-CH_3 (-0.10 - 0.05 ppm).

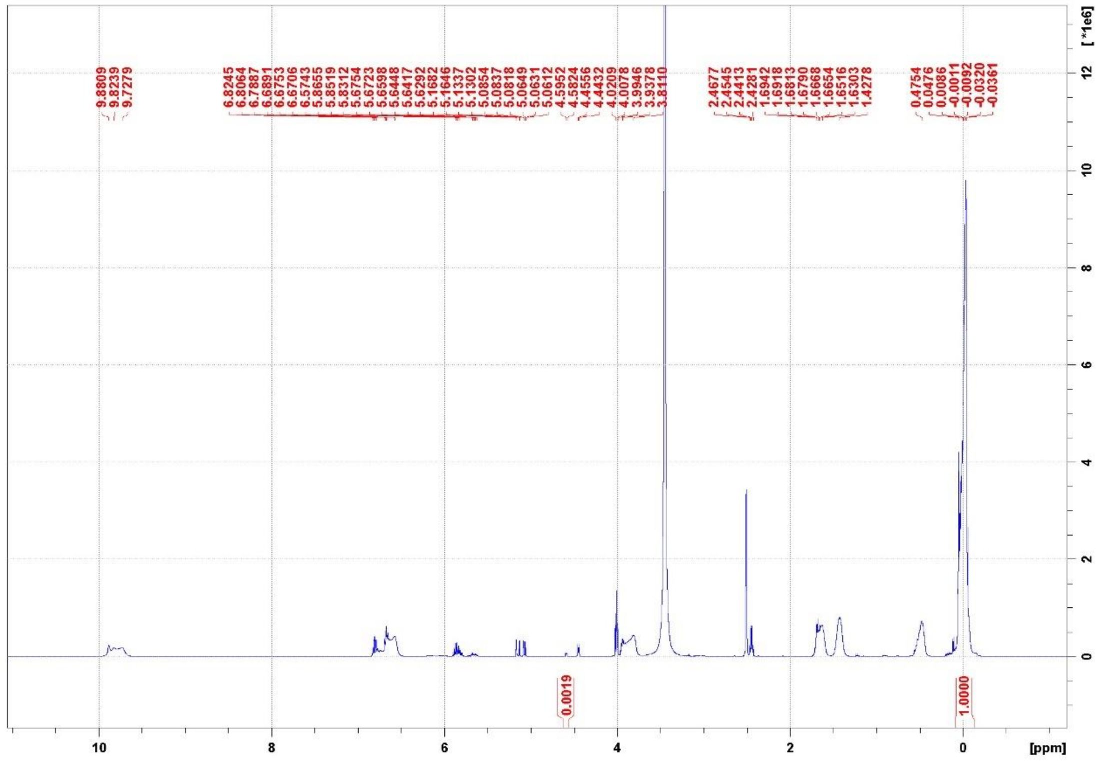

Figure S13. ^{1}H-NMR spectrum of PMFOS with integrated peaks of Si-H (4.57 – 4.61 ppm) and Si-CH_3 (-0.10 - 0.05 ppm).

Knowing that during the hydrosilylation the number of protons in the Si-H groups decreases and the number of protons in the Si-CH_3 groups remains unchanged, and knowing the initial content of monomers with Si-H groups in PMHS (average value 27.5%), the content of residual monomers with Si-H groups in PMFOS was calculated. The content of monomers with Si-H groups in PMFOS thus calculated is 0.76%. This means that the monomers with Si-H group were 97.2% converted during the reaction.
